# Electric Field Guided Assembly of One-Dimensional Nanostructures for High Performance Sensors

**DOI:** 10.3390/s120505725

**Published:** 2012-05-04

**Authors:** Devon A. Brown, Jong-Hoon Kim, Hyun-Boo Lee, Gareth Fotouhi, Kyong-Hoon Lee, Wing Kam Liu, Jae-Hyun Chung

**Affiliations:** 1 Department of Mechanical Engineering, University of Washington, Box 352600, Seattle, WA 98195, USA; E-Mails: devonb2@uw.edu (D.A.B.); jhkim78@uw.edu (J.-H.K.); hyunboo@uw.edu (H.-B.L.); gfotouhi@uw.edu (G.F.); 2 NanoFacture, Inc., P.O. Box 52651, Bellevue, WA 98015, USA; E-Mail: hoonlee@nano-facture.com; 3 Department of Mechanical Engineering, Northwestern University, 2145 Sheridan Road, Evanston, IL 60208, USA; E-Mail: w-liu@northwestern.edu; 4 World Class University (WCU) Program, School of Mechanical Engineering, Sungkyunkwan University, 300 Cheoncheon Suwon, 440-746, Korea

**Keywords:** review, electric field, nanowire, nanotubes, assembly, sensors

## Abstract

Various nanowire or nanotube-based devices have been demonstrated to fulfill the anticipated future demands on sensors. To fabricate such devices, electric field-based methods have demonstrated a great potential to integrate one-dimensional nanostructures into various forms. This review paper discusses theoretical and experimental aspects of the working principles, the assembled structures, and the unique functions associated with electric field-based assembly. The challenges and opportunities of the assembly methods are addressed in conjunction with future directions toward high performance sensors.

## 1. Introduction

The demands on sensor performance become very challenging with more ever more rigorous requirements for operating conditions, size, weight, and ease of use. Furthermore, a self-sustainable sensor system requires energy conversion, energy storage, and wireless transfer of measured parameters. In addition, various detection methods are required, such as electrical-, optical-, and other advanced imaging technologies. To meet these demands, nanowires and nanotubes are truly ideal candidates because one-dimensional nanostructures can be integrated into various devices such as single cell endoscopy [1], non-volatile memory [2], optical nanobarcodes [3], and nanoscale lights [3]. Multiple designs for transistors [4–9], logic circuits [2,10,11], and nanolasers [12,13] have also been developed.

Various assembly methods have been used to create these devices [5,7,8,14–17]. In each case, the precise placement of nanotubes and nanowires was essential to achieve the desired device performance. One of these methods, electric-field assembly, is often used to orient, attract, and assemble nanowires and nanotubes. When an AC electric field is applied to a medium including nanowires [18] or carbon nanotubes (CNTs), an electric dipole is induced to generate dielectrophoresis, which attracts and orients the nanostructures to electrodes [19]. In addition, electrophoresis has been applied to nanotube solutions to fabricate nanotube bundles [20]. Finally, a composite field has successfully demonstrated the capability to take advantage of both AC and DC fields [21,22].

These electric field assembly techniques have been used to create a wide variety of nanodevices in various configurations. At the smallest scale, individual CNTs [8,11] and nanowires [4,12] have been oriented and assembled into devices as electrical connections across electrodes [23–31]. Also, electric field induced methods have shown a great potential for the fabrication of nanoscale tips, fibrils, and fibers. These tips have been particularly useful for the production of high resolution AFM tips [32–37]. Also, methods such as electrospinning [38] and drawing [39] have increased the possible lengths of nanofibers.

On a larger scale, electric field guided assembly has also been used to align and embed bundles and arrays of nanotubes and nanowires into thin films[7] and bulk composites [40]. Thin films have been useful for various applications, including highly flexible electrodes [14], and organic LEDs [14]. Also, thin films made by layer-by-layer assembly [15,16] can facilitate new designs for photovoltaic devices, field effect transistors, nano-sized reactors, and drug delivery systems. Nanomaterials deposited in bulk products can be used to create novel materials with valuable mechanical, electrical, and optical properties. They are currently being used as fuel cell and super-capacitance electrodes, as well as bioactive coatings, sensors, and field emission devices [41].

This paper reviews the working principles and applications for the electric field guided assembly methods of nanotubes and nanowires. Toward the current and future demands, the opportunities and challenges of the assembly methods are discussed to encompass design of high performance nanostructured sensors.

## 2. Electric Field Guided Assembly of One-Dimensional Nanostructures

**Figure 1.**
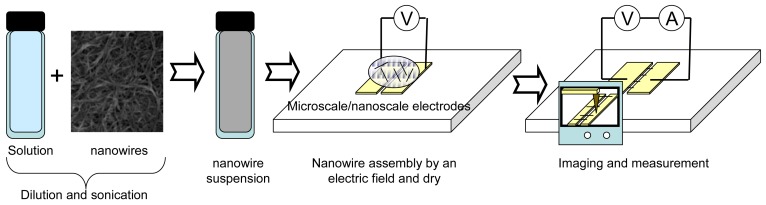
Conventional process of parallel assembly for nanowires using an electric field.

Figure 1 shows common procedure for nanowire assembly using an electric field. Synthesized nanowires are diluted in a solvent. A drop of the suspension is placed on microscale- and nanoscale electrodes where an electric field is applied. The nanowires in the solution are attracted and assembled on the electrodes. After the assembly, the solvent is evaporated in air. In the case of heavy molecular weight solvents, the solvent is removed at high temperature. The assembled nanowires are then characterized by microscopy and electrical measurement.

Electric fields can be used to manipulate nanowires in a solution in the form of electrophoresis, dielectrophoresis, and electroosmosis. Electrophoresis is generated by electrostatic force while dielectrophoresis is induced by the polarization of a nanowire in a medium. The direction and magnitude of the forces depend on the electric properties of particle and medium, and geometry of nanowires. Electroosmosis is generated by ions that interact with a charged surface. In this section, the details of electric field induced forces are discussed to understand the underlying mechanism and furthermore to envision a high yield assembly process of nanowires and nanotubes.

### 2.1. Electrophoresis

Electrophoresis is commonly used to position charged particles in a liquid. Nanowires are charged in a suspension solution and move toward the oppositely charged electrode. Electrophoretic force on a nanowire can be derived from the Coulomb's law [42]:

(1) $${\vec{F}}_{12}=\frac{Q_{1}Q_{2}}{4\pi {ɛ}_{0}r^{2}}{\vec{r}}_{12}$$

where *F⃗*_12_ is the force acting on the particle of charge 2 (*Q*_2_) by another particle of charge 1 (*Q*_1_), *ε*_0_ is the permittivity of vacuum, *r* is the distance between the particles, and *r⃗*_12_ is the unit vector from *Q*_1_ to *Q*_2_.

Electric field (*E⃗*) created by a point charge (*Q*) at the distance *r* is given by:

(2) $$\vec{E}=\frac{Q}{4\pi {ɛ}_{0}r^{2}}\vec{r}$$

By combining Equations (1) and (2), the electrophoretic force (*F⃗_EP_*) acting on a nanowire that has net charge *Q_net_* is:

(3) $${\vec{F}}_{EP}=Q_{\text{net}}\vec{E}$$

In Equation (3), the electrophoretic force can be computed by the net charge of a nanowire under an electric field.

**Figure 2.**
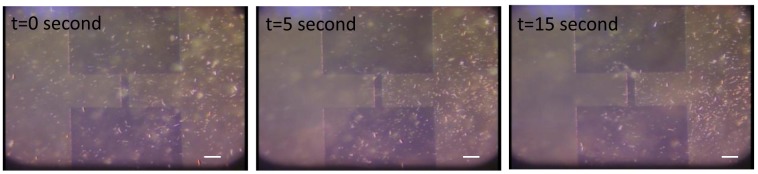
Electrophoretic deposition of Si nanowires; sequential images under a DC potential of 1.5 V. The scale bar is 100 μm.

To observe the electrophoretic behavior of nanowires, planar electrodes with a 25 μm gap were prepared. 15 μm-long nanowires were vertically fabricated on the Si wafer by using conventional photolithography and deep reactive ion etching. The Si nanowires were tapered from 300 nm to 1.2 μm. The Si nanowires suspended in dimethylformamide (DMF) solution were placed on the electrodes. When a DC potential of 1.5 V was applied on the electrodes, the nanowires were attracted to the edge and surface of the right electrode in Figure 2. The Si nanowires were negatively charged due to carboxyl ions in DMF and attracted to the positive electrodes due to electrophoretic force in 15 seconds.

### 2.2. Dielectrophoresis

Dielectrophoresis is induced by the dipole of a particle under a non-uniform electric field. The net force (*F⃗*) acting on the particle can be computed by summing the forces on each pole [42]:

(4) $$\vec{F}=Q\vec{E}(\vec{r}+\vec{d})-Q\vec{E}(\vec{r})$$

where *r⃗* is the position vector of negatively charged pole (−*Q*) and *d⃗* is the vector from negative pole (−*Q*) to positive pole (*Q*). In comparison with the characteristic length of a non-uniform electric field, *d⃗* is small. The first term in the right side of Equation (4) can be expressed by using Taylor series expansion:

(5) $$\vec{E}(\vec{r}+\vec{d})=\vec{E}(\vec{r})+\vec{d}\cdot \nabla \vec{E}(\vec{r})$$

where all the higher order terms are neglected by the assumption that the distance between the dipole is so small that their effect can be negligible. By applying Equation (5) into Equation (4), the force on the dipole (*F⃗_dipole_*) is obtained:

(6) $${\vec{F}}_{\text{dipole}}=Q\vec{d}\cdot \nabla \vec{E}(\vec{r})$$

For an AC field, the dipole moment (*p⃗* = *Qd⃗*) is:

(7) $$\vec{p}=V\alpha \vec{E}e^{i\omega t}$$

where *V* is the volume of a particle, *α* is the polarization factor (Clausius-Mossoti factor), and *ω* is the angular frequency of an input voltage. Time averaged dielectrophoretic force on a particle can be derived by using Equations (6) and (7):

(8) $$\langle {\vec{F}}_{\text{DEP}}\rangle =\frac{1}{4}V\text{Re}[\alpha ]\nabla {\left|\vec{E}\right|}^{2}$$

This analytical expression is called the effective dipole moment (EDM) theory. Using this equation, dielectrophoretic force on a particle can be computed by using a gradient of an electric field at a single point, e.g., particle center. However, EDM is not effective when a particle size is comparable to the characteristic length of an electric field. Because the force is computed at one point, the non-uniformity of electric field around a particle cannot be considered in the computation of the force.

For more rigorous approximation, DEP force can be calculated using the surface integral of the Maxwell stress tensor on the surface of a particle, which is Maxwell stress tensor (MST) theory [43,44]:

(9) $$\langle \sigma_{\text{MST}}\rangle =\frac{1}{4}{ɛ}_{m}\left(\vec{E}⊗\vec{E}*+\vec{E}*⊗\vec{E}-{\left|\vec{E}\right|}^{2}\text{I}\right)$$

(10) $$\langle {\vec{F}}_{\text{DEP}}\rangle =\oint \langle \sigma_{\text{MST}}\rangle \cdot \text{ndS}$$

where *ε_m_* is the permittivity of medium, E⃗* is the conjugate of *E⃗* and ⊗ is dyadic product. The MST is more complex, and requires more computation time than the EDM because the MST needs to solve a deformed electric field around a particle. However, the MST gives a more accurate result than the EDM.

**Figure 3.**
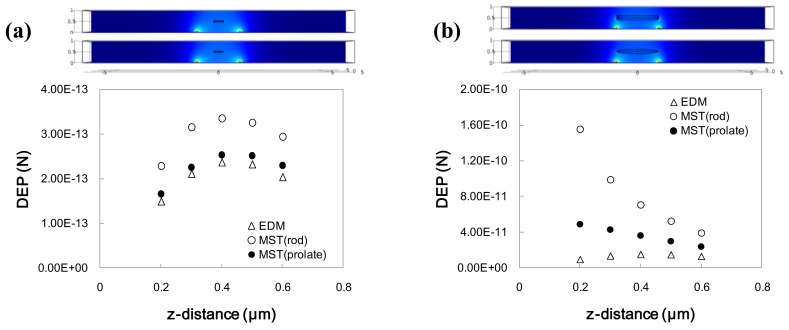
DEP force comparison between EDM and MST with different heights between a particle and electrode surface (**a**) Particle length is 0.5 μm; (**b**) Particle length is 2.0 μm.

To compare both EDM and MST methods, numerical analysis for a nanowire above a semicircular-shaped planar electrode was conducted. Figure 3 shows the DEP forces computed by EDM and MST.

The height of a nanowire from the electrode surface was varied. For the EDM method, the particle shape assumed was a prolate particle because of the limitation of the analytical expression. For the MST method, both prolate and cylindrical shaped particles were considered. Thus, the particle shape is identical for the prolate particles of EDM and MST. Nanowires of different particle sizes (L/H = 0.5/2 and 2/2; L: length of nanowire, H: gap between electrodes) with the same aspect ratio (D/L = 0.05/0.5 and 0.2/2; D: diameter of nanowire) were used for the comparison. Comparing Figure 3(a) and Figure 3(b), the computed DEP forces for the EDM and MST methods are quite different. Also the DEP forces vary according to the shape change because the surface charge on the particles varies.

The difference between the two methods can be mainly attributed to the EDM assumption that the distance between the dipole is so small that the higher order terms in Equation (5) can be neglected. However, as the particle size increases, the non-uniformity of the electric field increases, which increases the errors of the EDM due to the neglected terms.

The differences between EDM and MST are summarized in Table 1. In the EDM, the electric field in the entire domain (Ω) is solved by Laplace equation using only medium properties without considering frequency input. DEP force is simply calculated from the electric field norm at the particle center. The frequency dependency is applied by the Clausius-Mossoti factor. Note that this factor is dependent on the particle geometry, and thus can be given for simple geometries. On the other hand, complex Laplace equation in the solid domain (Ω*s*) and medium domain (Ω*m*) should be solved for the MST. DEP force is computed by the integration of Maxwell stress tensor over the particle surface. Therefore DEP on any arbitrary shape of particles can be computed by MST.

Considering the numerical results, the MST method should be used to predict the assembly of nanowires in order to fully consider the geometric effects of a nanowire. The EDM method tends to underestimate the DEP force acting on a nanowire. However, when a length of a nanowire is much smaller than a characteristic length of an electric field, the EDM method can be used.

**Figure 4.**
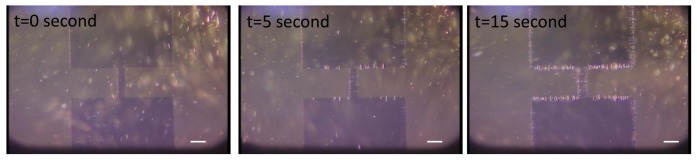
Sequential images of 15 μm-long nanowires deposition on the planar electrode using Dielectrophoresis. Applied voltage is 20 V_pp_ (peak to peak voltage) at 5 MHz. The scale bar is 100 μm.

Considering the DEP force and torque, nanowires are attracted to a high electric field region with orientation. More polarizable and longer nanowires can be more easily attracted, which can be used for the selective deposition of nanowires. For demonstration of DEP, 10 μL solution containing Si nanowires were placed on planar electrodes. When an electric potential of 20 V_pp_ at 5 MHz was applied between electrodes, Si nanowires were attracted and deposited along the edge of electrodes (Figure 4). The orientation of nanowires was orthogonal to the edge of electrodes.

### 2.3. Electroosmosis

**Figure 5.**
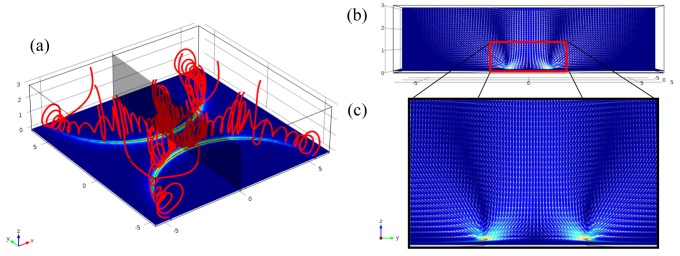
Simulation results of flow generated by the electroosmosis above the planar electrodes (**a**) Streamlines near the electrode edges; (**b**) Velocity vectors in the plane that indicated by the gray color in (a); (**c**) Magnified image of velocity vectors in vicinity of the edge.

When electric potential is applied to electrodes in a solution, ions are attracted to charged electrodes due to electrostatic interaction. The ions above an electrical double layer are not firmly anchored to the surface due to the electrical screening effect of the first layer. The ion concentration of this diffuse layer is changed along the variation of an electric field at the edge of electrodes. The decay of an electric field from the edge to the electrode surface is exponential, which induces ionic motion. The electroosmotic flow for a DC electric field can be calculated by [45]:

(11) $$U_{\text{EOF}}=-\frac{{ɛ}_{m}\zeta }{\mu }E_{t}$$

where *ζ* is the zeta potential of the layer, *μ* is the fluid viscosity and *E_t_* is the tangential directional electric field. Figure 5 is the simulation result for electroosmotic flow. Figure 5(a) shows the streamlines. Flow speed at the edge is the highest and decreases drastically far from the edge. Thus the rotational flow is induced above the electrode edge as shown in Figure 5(b,c). For an AC electric field, frequency dependent terms are included [46].

**Figure 6.**
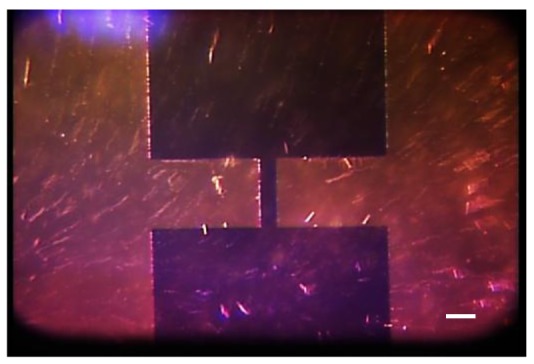
Behavior of 15 μm-long nanowires under electroosmosis. Applied voltage is 4 V_pp_ at 100 Hz. The scale bar is 100 μm.

For the electroosmosis observation, 10 μL solution containing Si nanowires were placed on planar electrodes. The rotation of Si nanowires was observed due to the electroosmotic flow (Figure 6). The electric potential was 4 V_pp_ at 100 Hz. The electroosmotic flow can attract nanowires in the vicinity of electrodes by circulation flows, which is much more effective than DEP. However, the random characteristic of the flow can be detrimental to precise assembly of nanowires. The attraction of ions can damage the surface of nanowires and electrodes.

## 3. Assembly of One-Dimensional Nanostructures

### 3.1. Planar Assembly

For sensor applications, nanowires and CNTs must be arranged reliably in desired locations and desired shapes. One way is to use electrodes to control the motion and attachment of nanowires and CNTs in specific structures. Dielectrophoresis has been used with both an AC and a DC field to align CNTs between electrodes [47]. Using these methods, the gap between two electrodes has been bridged by single nanowires or nanotubes, single bundles of nanowires or nanotubes, and arrays of nanowires or nanotubes. Once assembled, the nanowires could be characterized [48–51] and integrated into devices, such as field effect transistors and nanotube-based fluidic devices [52].

To control the number of nanowires between electrodes, voltage potential, frequency, and electrode shape should be controlled. In addition, deposition time and substrate material can affect the number of nanotubes attached between two electrodes [48]. Also, a self-limiting resistor, which decreases the electric field after a single CNT has been deposited, has been shown to increase the likelihood of the attachment of only one nanotube, instead of a full array [53]. Controlled deposition of a single CNT has also been accomplished through a composite AC biased electric field [21,22], real-time gap impedance monitoring [54], biased, floating potential electrodes and pre-defined trenches [55], and dielectrophoretic trapping [52]. Similar studies have also been conducted using metallic nanowires [53].

Single bundles of CNTs have also been selectively deposited on electrode pairs. The electrical and mechanical properties of these connections can be more easily predicted because the bundles have the average properties of all the nanotubes. Assembling these bundles across electrodes has enabled additional research into the current-voltage characteristics of CNT bundles [24], as well as their potential use as gas sensors and electronic devices [56]. Krupke *et al.* found that one of the best ways to assemble a single nanotube bundle across an electrode pair is to choose an electrode substrate that interacts strongly with CNTs, such as Ag [57]. Other methods include diluting the solution of nanotube bundles [58,59], changing the electric field strength [48,56], changing the probe geometry [58], and changing the electric field distribution [56,59].

Finally, networks of nanotubes and nanowires have been assembled between electrodes to produce devices such as gas- [60–63], thermal- [64], humidity- [65], and bio- [66] sensors, as well as UV photosensors [67], field effect transistors [5,68–70], and electronic devices [71]. In addition, because of their unique structures, these networks have also been used as filters to trap polystyrene microparticles [72] and light-emitting nanofiber arrays [73].

Though most work has been focused on producing devices, research has also been performed to investigate the behavior of CNTs and nanowires under different conditions. For instance, it was found that AC dielectrophoresis that is induced by a square wave can be a useful tool to align and attach multi-walled CNTs [25]. The square wave also decreased the number of impurities in the network of MWCNTs. In addition, the surface of single walled carbon nanotubes (SWCNTs) has been modified with octadecylamine in order to reduce entanglement and increase uniformity of nanotube density within a solvent [27].

**Figure 7.**
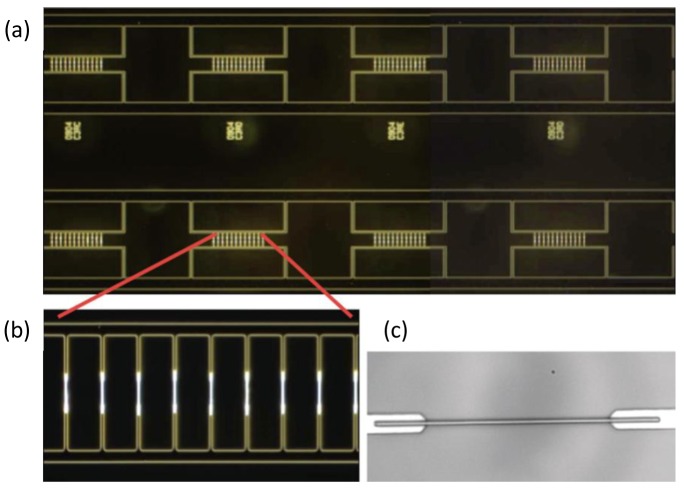
Optical images of Si nanowires assembled onto electrodes on a 4-inch quartz substrate. (**a**) Dark-field image of defect-free nanowire assembly on eight multi-finger electrode arrays; (**b**) Precision alignment of nanowires on a single, dense array. The electrode gap is 12 μm; (**c**) Nanowire on a single electrode (the gap is 12 μm). Reproduced from [81].

Multiple parameters, including the concentration of nanotubes and nanowires [59,74], electric potential [26,30,31,74–76], load resistance [26], frequency [26,29,74,75,77], electrode geometry [26,59,74,75,78,79], electric field dispersion [29,59], electric field type [31], deposition time [28,30,54], and suspension solution [26,27,75] have been used to change the density and geometry of these networks. Changing these quantities also affected the assembly time and the efficiency of the fabrication technique [76]. In particular, dielectrophoretic assembly combined with fluid flow could offer a high yield placement of nanowires [80]. This approach combines an AC electric field and shear flow in a microfluidic channel for the precise assembly and orientation of nanowires, which improves the deposition yield due to a continuous supply of nanowires. The uniform shear flow transports individual nanowires to the vicinity of electrodes, which can compensate the small magnitude of the dielectrophoretic force. This approach has been further developed to have a high deposition yield of 98.5% for Si nanowires (Figure 7) [81].

### 3.2. Fibril Assembly

**Figure 8.**
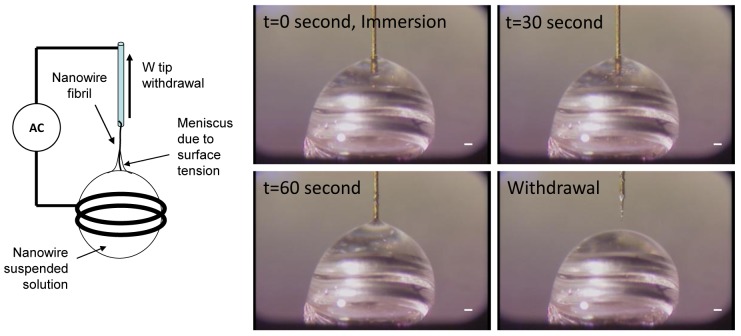
Sequential images of 15 μm-long nanowires one-dimensional assembly by using tungsten (W)-tip using dielectrophoresis. Applied voltage is 20 V_pp_ at 5 MHz. The scale bar is 100 μm.

One-dimensional assembly [82,83] is used to produce a tip, a fibril, or a fiber made of nanowires by combining an electric field and capillary action. Figure 8 shows a one-dimensional assembly process. Gold plated tungsten (W)-tip 50 μm in diameter was immersed in a DMF solution containing Si nanowires. An electric field of 20 V_pp_ at 5 MHz was applied to the W-tip and the ring that held a 2 μL solution drop. After 1 minute of immersion time, the W-tip was withdrawn gently from the drop. Due to dielectrophoretic attraction and capillary action, a small fibril was formed at the end of the W-tip, which is one-dimensional assembly.

The assembled structures are often used as bio and chemical sensors [32,33,84], force transducers [85], different types of beam emitters [32], mass sensors [32,85], linear bearings [32], nanomanipulation devices [32,33], and nanoelectromechanical switches [32,85]. CNTs are also often assembled onto atomic force microscope (AFM) tips [32–37]. In general, CNTs are the perfect material for AFM tips because of their small diameter, high aspect ratio, resistance to wear, and high resilience [33,34,36,37]. Also, in addition to topographical measurements, CNTs can measure electric and magnetic forces, surface potential, and frictional forces in a variety of environments including a vacuum, the ambient environment, and fluid environments [37]. Specifically, SWCNTs have been utilized as AFM tips because of their incredibly small size and their ability to get sub-nanometer resolution [37]. It was also suggested that a double walled carbon nanotube (DWCNT) makes the best AFM tip because it combines a longer shelf life with a high resolution image [37]. Multi walled carbon nanotubes (MWCNTs) have been also chosen because of the more stable structure [37]. Also, bundles of MWCNTs can be used to measure deep trenches [33,34].

There are several different methods used to assemble CNT nanotips. One method uses dielectrophoresis to attach nanotubes between a pair of asymmetric electrodes, a knife edged electrode and a flat electrode [32]. This type of assembly is very similar to the electrode assembly discussed above, however, the electrode assembly time is limited so that no networks or bundles are formed between the two electrodes. Once CNTs are assembled onto the knife electrode, individual nanotubes can be removed using a nanomanipulator system and then placed into tip-based nanodevices [32]. For this study, the average length of MWCNT tips was 400 nm, and over 60% of the MWCNTs were correctly aligned within 30° of the axis of the tip [32]. However, this type of manual manipulation can be very time-consuming [35].

Another common method of nanotip assembly is the application of an electric field in between a base electrode and a prefabricated tip [33,36]. In this assembly technique, the variables that affected tip assembly were tip and electrode orientation [33], frequency, concentration of the CNT solution, magnitude of induced voltage, and the shape of the prefabricated tip [36]. Park *et al.* reported a maximum yield of 75% after optimal conditions had been found [33]. In comparison with the manual nanomanipulator tip assembly method, described above, electric field tip assembly is faster and has a higher yield [33]. Potential disadvantages of this assembly method include contamination from other particles in solution and the attachment of a bundled fibril instead of single MWCNTs [33]. However, it has been shown that using an AC electric field instead of a DC electric field can decrease the amount of impurities present on the tips, and can increase yield of aligned CNTs [32,36]. Also, choosing the correct electric field properties as well as the curvature of the prefabricated tip can greatly increase the yield of this process [36].

Finally, a third method for CNT tip production uses a prefabricated tip as a working electrode and a small metal ring, holding a MWCNT solution, as a counter electrode [35], as illustrated in Figure 7. In this method, an electric field is applied to the ring and the prefabricated tip, causing the nanotubes to be drawn to the tip. Then the tip is slowly removed from the solution to form a nanotip with controlled length and orientation [35]. Unlike the methods described above, this method focuses on assembling a bundle of CNTs. This method can also be used for the fabrication of longer nanofibrils.

It has also been found that the current passing through an attached nanotip increases as the number of attached MWCNTs increases [85]. This is a useful way to obtain real-time feedback on the assembly of CNTs by DEP, instead of using SEM imaging to determine the number of nanotubes. Numerical analysis has also been performed to determine what forces are dominant during tip assembly and which assembly parameters would be the most successful for nanotip assembly [34].

Long fibers can also be assembled by dielectrophoresis in two main assembly categories: finite length and continuous fibers. Finite length fibers are fabricated in between two electrodes, similar to the connections made in the electrode assembly section. However, in this assembly technique, the variables that must be controlled are the solvent suspending the dispersed nanoparticles, the type of nanoparticle dispersed, electric field strength, and concentration of nanoparticles [86]. Within the solution, dielectrophoresis causes the fibers to align along the electric field, and then van der Waals forces bind the nanoparticles to each other, connecting the two electrodes [86,87]. This means that the maximum length of the fibers is controlled by the distance between the electrodes [86]. It also means that the length of the fibers created by this method can be controlled [87]. Fibers made by this technique usually range from 1 μm to over 1 cm in length [87]. Another benefit of this production method is that multiple fibers can be made at one time, all with similar lengths and diameters [86,87]. However, if the electric field is too strong, the individual fibers will be concentrated into one thick fiber, spanning the electrode gap [86].

Continuous fibers are fabricated through an assembly process that does not limit their maximum length. This technique is similar to the third tip production method, described above. For continuous fiber production, an electric field is applied to a solution that includes nanoparticles. Then, a tungsten tip is inserted into the solution and withdrawn [38,39,83,88–90]. When the tungsten tip is inserted into the solution, dielectrophoresis attracts nanoparticles to the tip and then van der Waals forces secure them [89]. In the process, the capillary force causes the nanotubes to form a fiber [90].

Surfactants are a useful tool that have been used to separate nanoparticles in solution and increase the uniformity of nanoparticles in the extracted nanofibers [38]. The type of surfactant chosen can affect the maximum length of fiber that can be assembled. For instance, using sodium dodecyl sulfate (SDS) causes short SWCNT bundles to be attached to the tungsten tip, whereas using sodium dodecylbenzenesulfonate (NaDDBS) allowed for long, uniform, porous fibers to be assembled [38]. While using surfactants, the highest extraction velocity achieved was 0.5 mm/s, and the fabricated fibers had a tensile strength of 400 MPa and a conductivity of 355 S/cm [38].

The diameters of these fibers are dependent on drawing rate, electric field gradient, sharpness of tungsten tip, and the concentration of nanomaterials in the solution [89]. Controlling these characteristics allows for a significant amount of length and diameter control [89]. For instance, using a withdrawal speed of 0.85 μm/s, fibers over 3 cm in length can be achieved [39]. It has also been noted that fibers fabricated using a DC electric field have larger diameters than those fabricated using an AC field [89]. For application, shorter length fibers assembled through this method can be used as electron emission sources [38,90] and high resolution microscopy probes [89]. Longer fibers can be used to reinforce polymer composites and increase anisotropic properties [38,88,89], woven into fabrics [38], used as electrodes [89], and conducting fibers [89].

Hybrid nanofibers can also be fabricated using a variety of different nanomaterials. The benefits of using multiple nanomaterials include the integration of different material properties, an improved production rate, and increased fibril length. For example, silicon carbide nanowires and SWCNTs have been used to form a hybrid nanofiber that has a high thermal conductivity of SiC nanowires and the gas sensing properties of SWCNTs [83]. These nanofibers could be used as nanoscale electromechanical systems, biosensors, or semiconducting devices.

### 3.3. Thin Film Assembly

CNTs are frequently used in thin films for their superior mechanical, electrical, and chemical properties. CNTs can both be distributed throughout a polymer matrix and assembled into a network to create a film of pure CNTs. The density of CNTs in the film, the spatial layouts of the CNTs, the lengths of the CNTs, and their orientation within the thin film can affect the properties of a thin film [15].

Sheets of pure CNTs can be fabricated by several methods. One fabrication technique uses two electrodes to create an electric field that orients and attracts CNTs to form a network [15,91,92]. In comparison with two-dimensional assembly, this fabrication technique is usually less precise because a CNT network forms with an approximate density, not a precise connection of a single CNT. This fabrication technique can be used to fabricate suspended nanoscale beams [91], thin-film transistors [92], actuators [93] and transparent electrodes [92]. Also, AC dielectrophoresis can be used to enrich the content of metallic or semiconducting CNTs for different applications, such as photovoltaic devices [15]. The size of the CNT thin films is controlled by the gap size between the two electrodes.

In a similar way, CNT thin films with complex structures can be fabricated using wafers containing interdigitated electrode elements [94]. Each of these electrode pairs directs part of the suspended nanotube solution into a geometric shape, which becomes part of the pattern of the thin film [94]. This method has been used to create electrically conductive channels that could be used for nanosensors [94]. Although this fabrication method can create larger films than the previous method, the maximum film area is still restricted by the size of the wafer. In addition, the capillary induced forces may cause misalignment of nanowires [94]. CNT thin films can also be applied as coatings to electrodes by AC electrophoresis. CNT coatings can be used to increase the charge storage capacity and decrease the impedance of electrodes [95].

CNTs can also be aligned within a polymer matrix to form flexible, versatile thin films with unique properties. These thin films offer both sensing and actuating abilities [96]. The aligned nanotubes within the polymer matrix increase the anisotropy of the material and thus enhance the electrical conductivity of the thin film by 3–5 orders of magnitude, in comparison to randomly oriented thin films [96]. The degree of alignment in CNT-polymer hybrid thin films can be controlled by the magnitude, frequency, and application time of the applied electric field [97].

**Figure 9.**
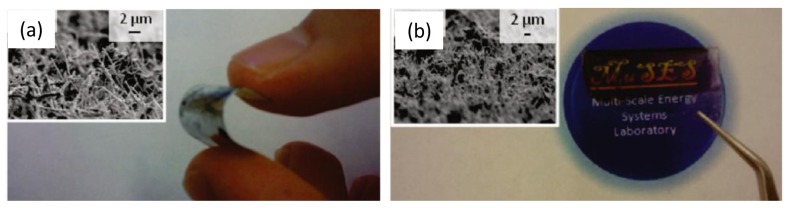
CNT forests were deposited on (**a**) flexible and (**b**) transparent substrates. Reproduced from [99].

Thin films of perpendicularly aligned CNTs have also been investigated for the distinct properties. High voltage electrophoretic deposition (HVEPD) can also be used to create high-quality, low deposition-time films [98,99]. HVEPD attracts and attaches CNTs perpendicular to an electrode surface through high voltage electrophoresis [98]. To decrease aggregation, a low concentration of nanotubes can be used [99]. After electrophoretic alignment, one group used ultrasonic irradiation to reassemble the nanotube bundles into a more precise organization [98]. The factors that influence HVEPD are the gap size between the two electrodes [98], concentration of nanotubes [99], deposition time [99], and the strength of the electric field [98]. It was also noted that short CNTs are more useful because they are easier to manipulate [98]. These thin films can be assembled on a variety of substrates including transparent, flexible, and stretchable materials, in addition to metal (Figure 9).

Thin films can then be formed into devices using the layer-by-layer (LBL) technique which builds different thin films on top of each other to form a functioning device [16]. This device assembly process allows for the inexpensive, versatile, bottom-up fabrication of nanodevices. In comparison with other techniques, LBL assembly can integrate a large variety of different materials and structures, and has the capability to make current devices much smaller. Also, LBL can be used in conjunction with many other assembly techniques including spin-coating, spraying, and photolithography, making it a versatile and useful assembly technique [16].

### 3.4. Bulk Assembly

Electric fields can also be used to align nanowires and nanotubes in bulk materials such as polymers, epoxies, or ceramics [40]. Electric fields are particularly useful for scalable production [100]. The bulk assembly process follows the stages of pre-processing, dispersion, alignment, and solidification. During pre-processing, epoxy matrices are poured [100], and polymer matrices are melted at high temperatures [40]. Then, during dispersion, nanoparticles are introduced to the matrix and evenly distributed, usually by mechanical stirring or sonication [40,101]. Next, an electric field is applied to enhance the alignment in the composite. AC fields generally produce an aligned final bulk product, whereas DC fields have been shown to produce branched networks of nanoparticles. Finally, the matrix material is cured to a solid state in the solidification stage. For best results, the electric field is continuously applied from alignment to complete solidification. It is common in this process for networks of nanoparticles to form along the electric field lines. These composites can be used as effective damping materials as well as more thermally stable and heat dissipative materials [102].

During this process, it was discovered that field strength and frequency played dominant roles in the degree of alignment [100,101,103]. While raising the field strength decreases the amount of time for orientation to occur, raising the frequency increases the amount of orientation and decreases the electrical resistivity of the composite [100]. To reduce the processing viscosity, materials like thermosets are often chosen to act as matrix materials [100]. Also, the nanowires introduced into the matrix can play a major role in the degree of orientation. Finally, vacuuming the final bulk composite to eliminate air bubbles can greatly increase the composite's strength [104].

Another bulk assembly method uses layers of poured epoxy to specialize the anisotropy of the composite. In this method, layers of epoxy are individually poured, aligned, and cured in the same method above. However, in between layers of epoxy the location, strength, and shape of the electrodes can be changed so that each layer is individually aligned in a different orientation [103]. This fabrication method provides greater control over the uniform properties of the bulk composite. Using this method, an epoxy's storage modulus was increased by approximately 50%, and the electrical conductivity, in the direction of nanotube alignment, was increased by four orders of magnitude.

Ceramic and metallic CNT composites can also be created through several different electrophoretic deposition techniques. If a layered coating is required, CNTs can be electrophoretically deposited onto an electrode first [41]. Then, ceramic or metallic particles can be introduced into the solution. These will then be electrophoretically deposited on top of the network of CNTs. If a more evenly dispersed layer is desired, the metallic or ceramic nanoparticles can be preassembled with CNTs in solution before electrophoretic deposition [41]. Also, if multiple layers are built up, a bulk metallic or ceramic nanocomposite can be formed. Materials that have been successfully used in this method with CNTs include SiO_2_, TiO_2_, MnO_2_, Fe_3_O_4_, hydroxyapatite, and bioglass. These composites can be used as fuel cell and supercapacitor electrodes, bioactive coatings, sensors, field emission devices, bioelectrodes, and photovoltaic films [41].

Though all of these methods work well, agglomerates often form due to van der Waals forces, causing some of the nanoparticles to be scattered in clumps around the composite instead of evenly distributed throughout the matrix [100,103,105]. This reduces the overall mechanical strength of the composites, though some groups have used agglomerates to increase the electrical properties of their final composites [101]. To reduce agglomeration, several methods have been invented, including chemical functionalization of CNTs [103], increasing cure time, and dispersing CNTs by acetone [104]. Chemically functionalized CNTs allow functional groups to adhere to the CNT surface, increasing the interaction between the CNTs and the matrix, as well as charging the surface of the CNTs to cause electrostatic repulsion. Dispersing the CNTs initially in acetone had two major outcomes. First, the addition of acetone in the polymer mixture decreased the viscosity of the polymer, allowing more CNT movement in the polymer. Second, the CNTs became electrostatically repulsive through the acetone processing.

Electrospinning is another useful assembly technique that can be used to produce a wide range of materials from fibers to thin films and bulk composites. These fibrous networks are useful because of their high surface area to volume ratio and quick fabrication time [106]. Also, electrospinning can be used with a wide variety of materials (including almost one hundred different polymers), can produce a large number of different morphologies, and can easily be scaled for larger applications. In the electrospinning process, polymers are either dissolved in solvents or heated into melt solutions and are subsequently placed into a capillary tube connected to a pipette that has a small diameter [106]. Then, a high voltage is applied to procure a charged jet of solution, which accumulates on a metal collecting plate [106,107]. As the solution jet travels to the collection plate, the polymer undergoes a stretching process and the solvents used to dissolve the polymer are evaporated [107]. Finally, the polymer in the fiber solidifies. In the case of polymer melt solutions, this process must occur within a vacuum [106]. As this process continues, a mat of nanofibers, whose diameters can range from less than three nanometers to greater than one micrometer, is assembled. Also, electrospun fiber networks can be created through the addition of nanoparticles, such as CNTs, into the polymer solution or the combination of two or more polymer solutions [106,107].

**Figure 10.**
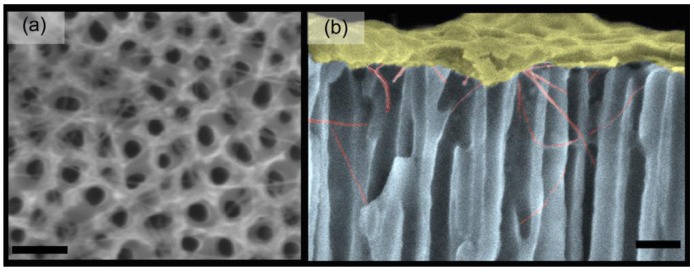
SEM micrographs of assembled SWCNT in anodic alumina array: (**a**) top view and (**b**) cross-sectional view. The top layer at this colored picture is the sputtered Au for imaging purposes. The scale bars are 120 nm. Reproduced from [108].

CNTs have also been recently used to create three-dimensional (3D) nanostructures by several different fabrication techniques. In one technique, electric fields have been used in conjunction with nanostructured templates, made out of anodic aluminum oxide, to direct the growth of large-scale 3D CNT structures (Figure 10) [108]. These nanotemplates have vertical nanoscale cylindrical holes that can be used to assemble SWCNTs, which can be useful in various assembly applications. Also, 3D CMOS devices have been assembled by another fabrication technique that includes optical lithography, lift-off, and dielectrophoresis [109].

This assembly technique has also been used to create a promising thermal sensor with high sensitivity, low weight, and low power consumption [110]. This 3D dielectrophoresis process has a yield of 98.7%, and can be scaled for mass production. The methods for planar-, fibril-, thin film-, and bulk assembly are summarized in Table 2.

### 3.5. Selected Assembly Using Electric Fields

Electric fields can be used to sort CNTs based on their electrical properties and sizes. CNTs can also be purified from unwanted substances such as catalyst particles and amorphous carbon materials [111,112]. These techniques can be used prior to device fabrication in order to target specific CNTs and increase the yield of a CNT assembly. This process is incredibly important to achieve CNTs with uniform electric properties. However, these CNTs are commonly used for very different purposes in nanodevices. For instance, metallic CNTs are used as electrical leads in nano-sized circuits, whereas semiconducting CNTs are used in field effect transistors and sensors [113]. Also, the size of CNTs can affect the electrical properties the nanotubes possess [111] as well as their capability to perform as various types of sensors. Therefore, it is important to sort CNTs by lengths and diameters, prior to device assembly.

The most common method used for sorting CNTs based on electrical properties, utilizes SWCNT soot [113]. This soot is suspended in D_2_O solution and prevented from aggregation by the addition of SDS and a sonication process. Afterwards, the solution is centrifuged for four hours, decanted, and placed on a microelectrode array. An AC electric field at 10 MHz and 10 V_pp_ is applied to attach metallic CNTs to the electrodes by dielectrophoresis, leaving the semiconducting CNTs in the solution. During this method of sorting, the applied frequency is very important because metallic SWCNTs experience a positive dielectrophoresis force regardless of frequency, but semiconducting SWCNTs experience a negative dielectrophoresis force only at high frequencies [29]. Through this method, metallic or semiconducting CNTs can be separated. However, bundles of CNTs could not be effectively separated because the dielectrophoretic force of a single metallic CNT in a bundle is much stronger than the dielectrophoretic repulsion of multiple semiconducting CNTs [29,113,114]. In this study, these bundles caused the purity of the metallic CNTs to decrease to 80% [113].

In addition, capillary electrophoresis has been used to separate CNTs based on diameter or bundle size, as well as length [111,115]. During capillary electrophoresis, a long, thin capillary tube, approximately 75 μm inside diameter and 1 m in length, is conditioned for two minutes with a 0.2 M NaOH solution [115]. Then the tube is flushed with deionized water and a buffer solution of 50 mM trizma base in 0.5% SDS solution. Then, the CNT solution, which has also been mixed with SDS to decrease aggregation, is introduced to the capillary tube. At this point, the CNT solution is pressurized at 100 mbar for thirty seconds and then a 15 kV separation voltage is applied [115]. In this method, the CNTs are separated because the total charge applied on the nanotubes is proportional to their surface area [111], which affects their electrophoretic mobility. CNTs with greater charge densities will be able to move more effectively. This method can also be used to separate individual nanotubes from bundled nanotubes.

Finally, electric fields can be used to purify CNT solutions from unwanted debris such as catalyst particles and amorphous carbon materials [111,112]. In one study, a set of gold microelectrodes in a microfluidic channel were used to purify CNTs [116]. The electric field used during this experiment had a voltage of 0.2 V_rms_/μm and a frequency of 1 kHz [116]. This frequency was chosen because it allowed the negative ions surrounding the carbon impurities to slip off, which forced the impurities to follow the electric field vectors perpendicular to the microelectrodes [19,116]. Once the carbon impurities moved close enough to the microelectrodes, they became attached by van der Waals attraction. Also, the voltage chosen allowed for the greatest ratio of impurities filtered out to final MWCNT output. However, some MWCNTs became attached to the microelectrodes during this process and were sacrificed [116]. With a higher voltage, more carbon impurities can be filtered from the solution, however the final yield of MWCNTs will also decrease [116]. This purification process is a useful way to increase the final yield of other assembly processes and enable the measurement of electronic properties of CNTs in regards to their physical size and shape [19]. It is also incredibly useful when the deposition of an individual CNT, across an electrode gap, is desired [117].

Dielectrophoresis can also be used to sort nanowires upon the lengths [80]. Under dielectrophoresis, the length of the deposited nanowires across a gap was dependent on the gap size; the nanowires, whose lengths were comparable to a gap size, were selectively deposited across electrodes. In other words, the nanowires much smaller than or larger than a gap size were not attracted to the gap because of the lower dielectrophoretic force. This result supports a prediction [43] that the dielectrophoretic force varies according to the ratio of the gap size to the nanowire length. The dielectrophoretic force is reduced when the gap size is much smaller than a nanowire length because the major polarizable direction is changed from the axial direction of the nanowire to the radial direction. Dielectrophoretic force also decreases when a gap size is much larger than a nanowire length because the electric field gradient around the nanowire is reduced at a larger gap. The maximum dielectrophoretic force is found when the gap size is 0.8 times the nanowire length. This result shows that the length of the deposited nanowires is determined by the gap size in dielectrophoretic assembly. The sorting methods using electric fields are summarized in Table 3.

## 4. Applications toward High Performance Sensors

The various fabrication methods described above are currently being used to create a variety of nanosensors, including biosensors, chemical sensors, gas sensors, thermal sensors, strain sensors, photosensors, and humidity sensors. Because of their small size, these devices are easy to incorporate and extremely sensitive. Currently, sensors have been created from one-dimensional assembly [17], hybrid nanowires [83], thin film assembly [15], and 3D assembly [110]. The variety of techniques for sensor assembly and optimization has greatly contributed to their success in laboratory studies. Through continued improvement, these devices can be integrated into future commercial products and processes.

One of the most interesting advances in the field of nanosensors has been the development of nanobiosensors [3,15,17,41,74,83,118]. These nanodevices have the potential to revolutionize the field of biosensors because their small size allows them to interact with individual molecules [15]. Currently, one-dimensional nanobiosensors are being used to detect cancer marker proteins in blood [17]. If correctly identified and measured, these biomarkers can be extremely useful for patient diagnosis and clinical management. Nanobiosensors have also been used to directly detect DNA strands and virus particles. These sensors could be used clinically, for disease detection, and commercially, for food safety.

Also, CNTs have been used in various gas [3,41,52,60,61,63,66,119,120] and chemical sensors [3,15,56] because many compounds can easily attach to the π bonds on the exterior of the CNTs, therefore changing the resistivity of the tube. This small electrical change can be easily recognized and quantified, allowing these sensors to have extremely large detection limits close to a few parts per billion [15]. In comparison with current sensor technology, CNT sensors demonstrate faster responses, higher sensitivity, and lower operating temperatures for a wide variety of gases [60,67]. These nanosensors have been used to detect hydrogen, ammonia, nitrogen dioxide, peroxide, and inorganic vapor [61,119]. Also, by aligning the CNTs and heating the sample, the sensitivity of these nanosensors can be enhanced [61]. However, there are several challenges associated with the assembly of chemical and gas sensors. First, producing a device that differentiates between complex chemicals and gas mixtures is still a challenge, though research into CNT functionalization has attempted to address this problem [15]. Second, assembling only CNTs without defects and impurities can be difficult [119].

CNTs can also be used to create thermal sensors that are small, lightweight, and highly sensitive [110]. CNT thermal sensors consume very little power and can be produced in high yield batches [64]. Considering the high yield of the process, these sensors could be used in multiple industries including biomedical, automobile, and aviation [110]. However, because some of these sensors are built using new 3D assembly techniques, more research is necessary before these devices can be produced commercially.

Finally, SWCNTs have been used in elastomeric matrixes to fabricate strain sensors [15]. When placed under strain, the CNTs in the elastomer deform and change their electronic properties due to changes in the band gaps and/or connections between the CNTs [15]. As the strain is released, this electronic change is reversed [15]. Many of these networks include randomly oriented CNT networks, however, aligned networks with “wavy” structures have proven to be more effective [15]. These strain sensors are capable of accommodating strains of greater than 20% [15].

In addition to CNTs, ZnO nanowires have also been used in the fabrication of several types of sensors. First, ZnO nanowires have been integrated into photosensors because of their high surface area to volume ratio, which increases UV light adsorption and the sensitivity of these sensors [66,67]. When assembled across electrode gaps, these nanowires are aligned along the electric field lines [67]. Then, once exposed to UV light, the conductance of these nanowires exponentially increases and can be detected [67]. However, the response time for ZnO nanowires to UV light is slow, possibly due to the adsorption of ambient gas molecules [66,67]. If this is the case, a purification process may accelerate the response time. ZnO nanowires have also been used to fabricate a humidity sensor [65]. This sensor utilizes the high specific area, fine particle size, and quantum confinement properties of ZnO nanoparticles. The alignment of the ZnO nanowires through dielectrophoresis was crucial to the sensitivity of the humidity sensor [65].

Though these sensors offer promising advances for a variety of fields, there are still several challenges associated with their assembly. First, many nanosensors are difficult to manufacture quickly and consistently in large quantities [17,118]. Second, the high cost of nanomaterials and nanoassembly prevent some nanosensors from being commercially produced [17]. Third, many nanosensors are currently not robust enough to be used clinically or commercially [118]. Finally, further testing must be conducted to determine potential toxicology and environmental regulations [17].

## 5. Summary

In the last decade, various types of sensors have been fabricated by using an electric field to assemble one-dimensional nanostructures, including nanotubes and nanowires. In addition to sensing, various functions including energy storage, energy conversion, electronics, imaging, and detection have been demonstrated to offer an enormous potential for developing a self-sustainable sensor system. One-dimensional fibrils, two-dimensional structures, thin films, and bulk structures have been integrated into devices through electric field assembly. Compared with other assembly methods, the major contribution of an electric field is purification, alignment, and precise assembly. In addition, the integrated devices are compatible with biomaterials and biosensors due to their small size, which fosters numerous bioapplications. The remaining challenges for commercialization are reduction of manufacturing cost, realization of scalable manufacturing, reliable performance of sensors, long-term shelf life of devices, and packaging.

## Figures and Tables

**Table 1.** Comparison between EDM and MST.

**Method**	**EDM**	**MST**
**Electric Field**	∇·(*σ_m_*∇*V*) = 0, in Ω*E* = −∇*V*	∇·(σ_m_*∇*V*) = 0 in Ω*_m_*∇·(σ_s_*∇*V*) = 0 in Ω*_s_E* = −∇*V*
**DEP Calculation**	$\langle {\vec{F}}_{\text{DEP}}\rangle =\frac{1}{4}V\text{Re}[\alpha ]\nabla {\left|\vec{E}\right|}^{2}$	$\begin{array}{l} \langle \sigma_{\text{MST}}\rangle =\frac{1}{4}{ɛ}_{m}\left(\vec{E}⊗\vec{E}*+\vec{E}*⊗\vec{E}-{\left|\vec{E}\right|}^{2}\text{I}\right)\\ \langle {\vec{F}}_{\text{DEP}}\rangle =\oint \langle \sigma_{\text{MST}}\rangle \cdot \text{ndS} \end{array}$
**Shape Dependency**	Spherical, oblate, and prolate geometries	Arbitrary shape

**Table 2.** Summary of electric field-induced assembly methods.

**Assembly Methods**	**Nanowires or CNTs**	**E-Fields**	**Main Mechanism**	**Applications**	**Schematic**
Planar assembly	Individual or bundles	AC and DC fields	Electrophoresis, dielectrophoresis, and fluid-flow	Gas-, thermal-, humidity- and bio- sensors, UV photosensors, and FET	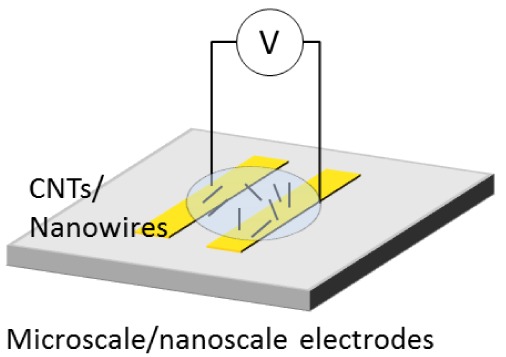
Fibril assembly	Individual or bundles			Bio, chemical sensors, force transducers, mass sensors, and AFM tips	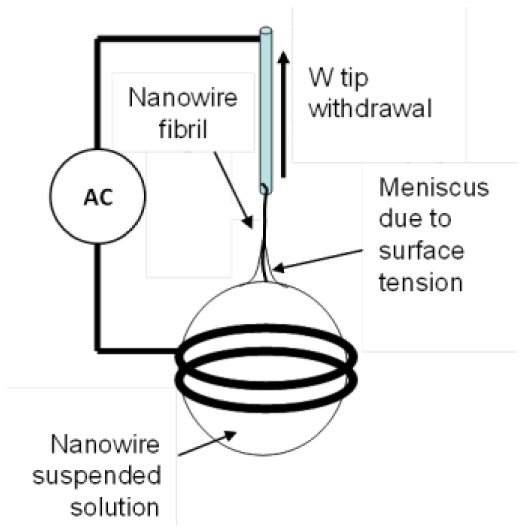
Thin film assembly	Bundles		Electrophoresis and dielectrophoresis	Nanoscale beams, thin-film transistors, actuators, and transparent electrodes	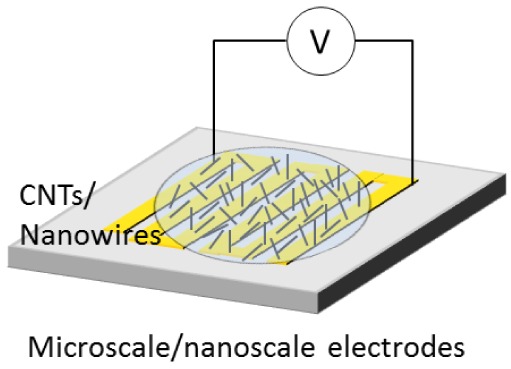
Bulk assembly	Bundles in polymer matrix		Electrophoresis and dielectrophoresis in polymer matrix with thermal curing	Fuel cells, super capacitor electrodes, bioactive coatings, bioelectrodes, sensors	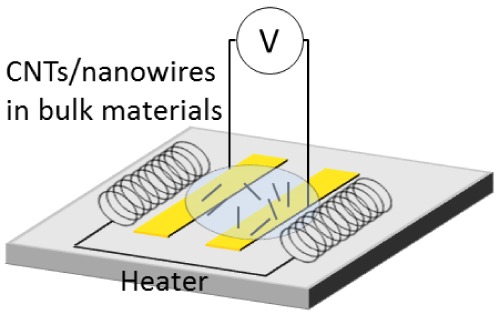

**Table 3.** Summary of selected assembly methods using electric fields.

**Sorting Methods**	**Electric Fields**	**Main Mechanism**	**Purpose**
Electric properties	AC electric field	Dielectrophoresis	Separation of metallic CNTs out of metallic and semiconducting CNTs
			Purification of CNTs out of mixture of CNTs, amorphous carbon, and other particles including catalysts
Electrophoretic mobility	DC electric field	Electrophoresis	Sorting based on lengths, diameters, and other dimensions
Gap sizes between electrodes	AC electric field	Variation of dielectrophoretic force of nanowires upon a gap size	Sorting based on polarizable lengths of dielectrophoresis
